# Rho‐associated coiled‐coil kinase 1 activation mediates amyloid precursor protein site‐specific Ser655 phosphorylation and triggers amyloid pathology

**DOI:** 10.1111/acel.13001

**Published:** 2019-07-09

**Authors:** Yong‐Bo Hu, Ru‐Jing Ren, Yong‐Fang Zhang, Yue Huang, Hai‐Lun Cui, Chao Ma, Wen‐Ying Qiu, Hao Wang, Pei‐Jing Cui, Hong‐Zhuan Chen, Gang Wang

**Affiliations:** ^1^ Department of Neurology Neuroscience Institute Ruijin Hospital Affiliated to Shanghai Jiao Tong University School of Medicine Shanghai China; ^2^ Department of Pharmacology and Chemical Biology Shanghai Jiao Tong University School of Medicine Shanghai China; ^3^ National Clinical Research Centre for Neurological Diseases Beijing Tiantan Hospital Affiliated to Capital Medical University Beijing China; ^4^ Faculty of Medicine, Neuroscience Research Australia UNSW Australia Sydney New South Wales Australia; ^5^ Department of Human Anatomy, Histology and Embryology, Institute of Basic Medical Sciences, Neuroscience Center, Chinese Academy of Medical Sciences, School of Basic Medicine Peking Union Medical College Beijing China; ^6^ Department of Geriatrics Ruijin Hospital Affiliated to Shanghai Jiao Tong University School of Medicine Shanghai China; ^7^ Institute of Interdisciplinary Science, Shuguang Hospital Shanghai University of Traditional Chinese Medicine Shanghai China

**Keywords:** Alzheimer's disease, amyloid precursor protein, phosphorylation, Rho‐associated coiled‐coil kinase 1

## Abstract

Rho‐associated coiled‐coil kinase 1 (ROCK1) is proposed to be implicated in Aβ suppression; however, the role for ROCK1 in amyloidogenic metabolism of amyloid precursor protein (APP) to produce Aβ was unknown. In the present study, we showed that ROCK1 kinase activity and its APP binding were enhanced in AD brain, resulting in increased β‐secretase cleavage of APP. Furthermore, we firstly confirmed that APP served as a substrate for ROCK1 and its major phosphorylation site was located at Ser655. The increased level of APP Ser655 phosphorylation was observed in the brain of APP/PS1 mice and AD patients compared to controls. Moreover, blockade of APP Ser655 phosphorylation, or inhibition of ROCK1 activity with either shRNA knockdown or Y‐27632, ameliorated amyloid pathology and improved learning and memory in APP/PS1 mice. These findings suggest that activated ROCK1 targets APP Ser655 phosphorylation, which promotes amyloid processing and pathology. Inhibition of ROCK1 could be a potential therapeutic approach for AD.

**Abbreviations**
AD: Alzheimer's disease
AICD: APP intracellular domain
APP: amyloid precursor protein
BACE1: beta‐secretase 1
Co‐IP: co‐immunoprecipitation
CTF: C‐terminal fragments
ELISA: enzyme‐linked immunosorbent assay
GFP: green fluorescent protein
MAPK: mitogen‐activated protein kinase
MS: mass spectrometry
MWM: Morris water maze
NSAIDs: nonsteroidal anti‐inflammatory drugs
PLA: proximity ligation assay
PTM: post‐translational modification
PVDF: polyvinylidene flouride
RLU: relative luminescence unit
ROCK1: Rho‐associated coiled‐coil kinase 1
WT: wild‐type

## 1 INTRODUCTION

Alzheimer's disease (AD) is the most prevalent form of dementia characterized by progressive cognitive decline (Goedert & Spillantini, 2006). The disease is characterized by β‐amyloid (Aβ) deposition, neurofibrillary tangles (NFTs), and neuron loss. Among those pathological changes, the extracellular accumulation of amyloid plaques in brain derived from amyloid precursor protein (APP) cleavage is considered to be a specific hallmark of AD. However, as a type I transmembrane protein, the physiological function of APP remains unclear (Deyts, Thinakaran, & Parent, 2016). Two principal APP proteolytic processing pathways occurring in a competitive manner have been well characterized: an α‐secretase‐mediated nonamyloidogenic pathway and a β‐secretase‐mediated amyloidogenic pathway (O’Brien & Wong, 2011). Generally, α‐secretase or β‐secretase cleaves APP extracellular domain and generates soluble N‐terminal fragments sAPPα or sAPPβ, as well as C‐terminal fragments (CTF) α‐CTF and β‐CTF, respectively. α‐CTF or β‐CTF will further be cleaved by γ‐secretase, releasing the P3 peptide or Aβ and the APP intracellular domain (AICD) into the cytoplasm^3^. Among these, APP cytoplasmic CTFs are the most active domain, harboring capacity to interact with macromolecules such as caspase‐3, mitogen‐activated protein kinase (MAPK), and protein kinase C (PKC; Pastorino et al., 2012). Additionally, the APP‐CTF domain may be involved in intracellular signaling transduction and regulation of ion channels and Ca^2+^ concentration (Lacampagne et al., 2017). However, the roles of APP‐CTF in AD pathogenesis are not fully understood. One of the major mechanisms by which the APP‐CTF is modeled involves direct modification by phosphorylation (Lee et al., 2003; Vingtdeux et al., 2005).

Rho‐associated coiled‐coil kinase 1 (ROCK1) functions as a versatile kinase, phosphorylating various substrates such as myosin‐light‐chain phosphatase, LIM kinase, phosphatase and tensin homologue, insulin receptor substrate, ezrin/radixin/moesin proteins, and JNK‐interacting protein (Liu et al., 2013; Peng et al., 2016; Surma et al., 2014). Additionally, previous studies found that ROCK1 could regulate Aβ and its expression was increased in the brain of AD patients (Henderson et al., 2016). Statins as well as nonsteroidal anti‐inflammatory drug (NSAID) could curb ROCK1 activity and activate sAPPα shedding, showing potential for AD prevention (Hu et al., 2016; Pedrini et al., 2005; Zhou et al., 2003). However, the probable relation of APP with ROCK1 and potential roles for ROCK1 in APP modification and metabolism remain unclear.

Therefore, the present study aims to investigate the ROCK1 activity and its interaction with APP in AD mouse model and patients, identify whether APP is a phosphorylation substrate of ROCK1, and confirm the subsequent effects of ROCK1‐induced APP phosphorylation on APP processing. Furthermore, we investigate the molecular mechanisms of APP phosphorylation through ROCK1 signaling and its promotion of the amyloidogenic pathway. Finally, we validate whether altering APP phosphorylation or ROCK inhibition could be a therapeutic option for AD.

## 2 METHODS AND MATERIALS

### 2.1 Mice and ethics statement

APP/PS1 mice and littermate control C57BL/6J were provided by the Model Animal Research Center of Nanjing University. The experiments conducted in this study had ethical approval from the Ethics Committee of Shanghai Jiao Tong University. Mice were anesthetized and transcardially perfused with ice‐cold phosphate‐buffered saline (PBS). One half of brain was dissected and homogenized for ROCK1 activity assay. The other half of brain was fixed in 4% paraformaldehyde overnight at 4°C and then incubated in 30% sucrose for immunofluorescence and proximity ligation assay.

### 2.2 ROCK1 kinase activity assay

Rho‐associated coiled‐coil kinase 1 activity assays were performed using the ROCK1 ADP‐Glo Kit (Promega) according to the instructions. Kinase reaction system contained 100 nM ROCK1, S6K substrate, and 1 mM ATP in 100 mM KCl, 20 mM Tris (pH 7.5), 0.05 mg/ml BSA, 1 mM TCEP, and 2 mM MgCl_2_. Cell lysates and tissue homogenates (30 μg) with the reaction solution were incubated for 40 min at room temperature. Luminescence (RLU) was measured using a Synergy MX Plate Reader (BioTek).

### 2.3 Cell culture and transfection

Human embryonic kidney HEK293 cells stably expressing human APP695 harboring the Swedish mutation (HEK293 APP695sw) and PC12 cells were maintained in DMEM (Gibco) with 10% FBS and 1% penicillin/streptomycin at 37°C in a 5% CO_2_ incubator. Cells were plated at 10^6^ cells/cm^2^ density in 6‐well dishes coated with 100 μg/ml poly‐lysine. Rho‐associated coiled‐coil kinase 1 over‐expressing plasmid (pCAG‐myc‐ROCK1myc‐727 Δ3, ROCK1 CA) was constructed according to a previous publication (Fujisawa, Fujita, Ishizaki, Saito, & Narumiya, 1996). For plasmid or small interfering RNA (siRNA) transfection, equivalent amounts of cells were plated, and transfections were performed using Lipofectamine 2000 according to the manufacturer's instructions. At 48 or 72 hr post‐transfection, cells were harvested and then processed for Western blot analysis or fluorescence imaging. For Aβ40 treatment, Aβ40 oligomers were added to PC12 cell cultures at a final concentration of 10 μM (Benseny‐Cases et al., 2018). After 72 hr incubation, cells were harvested for Western blot analysis.

### 2.4 Real‐time RT–PCR

Total RNA was isolated from brain tissue homogenates using TRIzol reagent according to the manufacturer's instructions (Sigma‐Aldrich). 2 μg RNA was used for reverse transcription to single‐strand cDNA. Gene expression of ROCK1 was quantified by real‐time RT–PCR. The primer for RT–PCR was as follows: forward: 5′‐GACTGGGGACAGTTTTGAGAC‐3′; reverse: 5′‐GGGCATCCAATCCATCCAGC‐3′. Results were calculated using the ΔΔCt method. Data were normalized to GAPDH expression.

### 2.5 Human brain tissue analysis

Postmortem brain tissues from six controls and six AD patients with clinical diagnosis and neuropathological confirmation (Figure S3) were obtained from the Human Brain Bank of Peking Union Medical College (PUMC). This study had ethical approval from ethical committees of PUMC and Shanghai Jiao Tong University.

### 2.6 Proximity ligation assay (PLA)

Proximity ligation assay was performed using Duolink PLA Kit (Sigma‐Aldrich) in 4% PFA‐fixed brain tissue or cells before permeabilization in 0.1% Triton X‐100 for 30 min. The in situ PLA detection kit 596 was used to visualize single‐molecule interactions for ROCK1‐APP or BACE1‐APP. Samples were blocked and incubated with specific primary antibodies at 4°C overnight. Secondary antibodies (anti‐rabbit PLUS and anti‐mouse MINUS probes) were added to the reaction and incubated for 1 hr at 37°C. The hybridized oligonucleotides were ligated for 30 min at 37°C. Then, amplification solution was added with polymerase and incubated for 100 min at 37°C. After mounted with mounting medium, the PL signal was visualized as a chromatic (red) spot and was captured by Confocal LSM 510 (Zeiss).

### 2.7 Mass spectrometry analysis

Synthetic APP‐CTF peptide (10 μM, AnaSpec) was incubated with recombinant active ROCK1 protein (10 μM, Abcam), 1 × NEB PK buffer, and 200 μM ATP for 3 hr at 30°C. Samples were digested with trypsin, and the resulting peptides were analyzed by reverse‐phase liquid chromatography coupled with tandem mass spectrometry (LC‐MS/MS) independently as previously described (Herskowitz et al., 2010).

### 2.8 Western blot

Brain tissue or cells were lysed with lysis buffer and subjected to a 15,000 *g* spin to remove nuclei and debris. Total protein concentrations were determined using the Enhanced BCA Protein Assay Reagent (Beyotime). Equal amounts (30 μg protein) were loaded onto SDS‐PAGE gels. Proteins were transferred onto polyvinylidene flouride (PVDF) membranes. Membranes were blocked with 5% dry milk solution for 1 hr and then incubated with primary antibodies overnight at 4°C. Membranes were washed with TBST, and protein bands were visualized using horseradish peroxidase‐conjugated species‐specific secondary antibodies. GAPDH was used as loading control. Images were captured, and band intensities were quantified using an Odyssey Image Station (LI‐COR).

### 2.9 Co‐immunoprecipitation (Co‐IP)

HEK293 cells were lysed with lysis buffer after 48 hr transfection. After clarification by centrifugation at 4°C for 30 min at 15,000 *g*, 500 μl supernatant was incubated with 20 μl of protein A/G agarose beads for 4 hr with gentle rotation at 4°C. The beads were washed four times with the cell lysis buffer, and precipitates were eluted with 2 × SDS‐PAGE sample buffer by boiling and then analyzed by Western blot for anti‐APP and anti‐ROCK1 immunoreactivity, respectively.

### 2.10 Immunofluorescence

Samples were fixed, and permeabilization was performed in PBS with 0.3% Triton X‐100 for 10 min at RT. After blocking, sections were incubated overnight at 4°C with primary antibody. After washing three times with PBST, the sections were incubated with Alexa Fluor 488‐conjugated donkey anti‐rabbit or Alexa Fluor 594 anti‐mouse IgG secondary antibodies (Invitrogen), respectively, and photographs were taken using Leica SP8 confocal microscope (Leica).

### 2.11 ELISA

Media were collected 72 hr after transfection, and cell debris was removed by centrifugation. To detect the concentration in brain lysates, mouse brains were homogenized and diluted with PBS. sAPPα, sAPPβ, and Aβ 40 were detected using sandwich ELISA kits (IBL) for human sAPPα, sAPPβ, and Aβ 40 following the manufacturer's instructions. Plates were read at 450 nm on a Synergy MX Plate Reader (BioTek).

### 2.12 Viral construct generation and injections

Human cDNA encoding APP^695^ or APP^695^ S655A with Swedish mutation was cloned into pLenti‐GFP vector. The lentiviral vectors coding for GFP were used as a control. Lentivirus vectors for ROCK1 shRNA expression were constructed. Before injections, lentiviral vectors were diluted with sterile PBS to achieve a titer of 1 × 10^8^. Mice were anesthetized by inhalation of 2.5% isoflurane. Using bregma as a reference point, coordinates for stereotaxic injections were set: −2.46 mm antero‐posterior; 0 mm lateral; and 2 mm dorso‐ventral from the skull. Mice were injected into the third ventricle with 5 μl of viral suspension. Two months after the injection of the viral vectors, the mice were subjected to behavioral tests and histochemical analysis.

### 2.13 Generation of antibody specific to APP pS655

Four rabbits were immunized with the peptide LKKKQYTS(p)IHHGVVE which included the 15 amino acids flanking APP^695^ four times, with 3‐week intervals between injections. The antiserum was purified by affinity chromatography and was then absorbed to a spanning peptide (LKKKQYTS(p)IHHGVVE). The antibody titers against the immunizing peptide were determined by an enzyme‐linked immunosorbent assay. The maximal dilution giving a positive response for horseradish peroxidase was 1:128,000 with chromogenic substrate. The immunoactivity of the antiserum was further confirmed by Western blotting and immunohistochemistry. The confirmatory data of APP pS655 antibody were provided in Figure S4.

### 2.14 Animal treatment with ROCK1 inhibitor Y‐27632

Seven‐month‐old APP/PS1 transgenic and WT male mice were kept with accessible food and water. For ROCK1 inhibition, Y‐27632 was administered intraperitoneally at a concentration of 20 mg/kg body weight every 3 days for 45 days according to previous studies with Y‐27632 in animal disease models (Shibata et al., 2003). A total of 12 APP/PS1 mice were used and 10 wild‐type mice served as a control group, receiving saline without supplementation (vehicle).

After Morris water tests, mice were deeply anesthetized and perfused with PBS, and their brains were removed. One half of brain was fixed in 4% paraformaldehyde in PBS and cryoprotected in 30% sucrose in PBS at 4°C until sectioning. The brain tissues were cut into sagittal 30‐μm sections. In the other half of brain, the cortex was dissected and homogenized for Western blot and ELISA.

### 2.15 Morris water maze (MWM)

After intervention, APP/PS1 transgenic and WT male mice were trained in a round water pool (1.1 m in diameter) with extra‐maze cues. Each subject was given four training trials per day for 5 consecutive days, to learn to find a hidden platform located 1.5 cm below the water surface. In each trial, mice were given 60 s to find the invisible platform in one of four different positions. The animals were allowed to stay at the platform for 10 s if they found the platform within the given time. However, if the animals failed to find the platform within the given time, they were manually guided to the platform and left there for 10 s. The escape latency, that is, the time required to find and climb onto the platform, was recorded for up to 60 s. After each trial, mice were dried and kept in a warm cage. The inter‐trial interval for each mouse was 10 min. For the probe trials, the platform was removed and the mice were allowed to swim for 60 s. The probe trials were conducted 24 and 48 hr after the last training trial. Data were recorded and analyzed using the automated tracking system.

### 2.16 Antibodies and reagents

Mouse rabbit anti‐APP (Novus, 1:10,000), rabbit anti‐APP‐CTF (Sigma‐Aldrich, 1:5,000), anti‐ROCK1 antibody (Abcam, 1:500), rabbit anti‐SP1 (Abcam, 1:1,000), rabbit anti‐SP6 (Sigma‐Aldrich, 1:1,000), mouse anti‐sAPPα (IBL, 1:50), mouse anti‐Aβ (Cell Signaling, 1:1,000), and rabbit anti‐BACE1 (Cell Signaling, 1:1,000) were used. Loading controls (GAPDH, Sigma‐Aldrich, 1:1,000) were used for Western blot standardization. Lipofectamine 2000 was sourced from Invitrogen. Y‐27632 was purchased from Abcam.

### 2.17 Statistical analysis

Statistical analyses were performed by Student's *t* test for two‐group comparisons, and one‐way or two‐way ANOVA followed by post hoc tests for multiple comparisons among more than two groups. The results were presented as Mean ± *SEM*, and *p* < 0.05 was accepted as statistically significant.

## 3 RESULTS

### 3.1 ROCK1 activity and its interaction with APP are up‐regulated in AD

**Figure 1:**
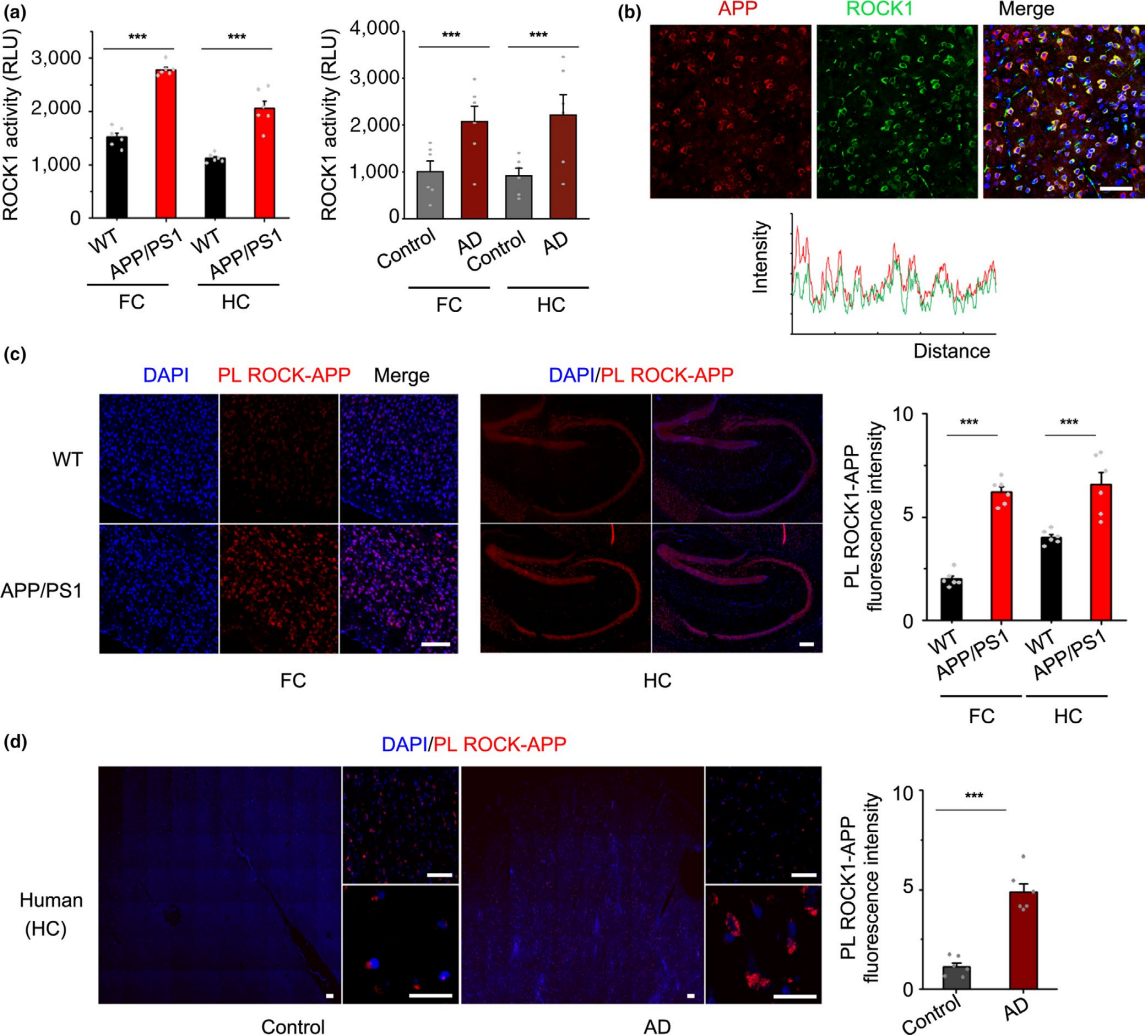
Up‐regulated ROCK1 activity and its interaction with APP in AD. (a) left panel, ROCK1 activity increased in the frontal cortex (FC, *t*_10_ = 4.64) and hippocampus (HC, *t*_10_ = 4.9) of 8‐month APP/PS1 mice relative to wild‐type control (WT) by kinase assay. right panel, ROCK1 activity increased in the frontal cortex (FC, *t*_10_ = 5.36) and hippocampus (HC, *t*_10_ = 4.67) of AD patients relative to normal control. (b) Co‐localization between APP and ROCK1 in the brain of APP/PS1 mice. Fluorescence intensity profiles are also presented, measured along the line marked in the fluorescent images. Scale bar, 50 μm. (c) Proximity ligation and quantification of PLA signal between ROCK1 and APP in the cortex *(t*_10_ = 18.94) and hippocampus (*t*_10_ = 6.33) of APP/PS1 and WT mic. Scale bar, 50 μm. (d) Proximity ligation and quantification of PLA signal between ROCK1 and APP in the hippocampus of AD patients and normal control, *t*_10_ = 8.68. Scale bar, 50 μm. Data were presented as Mean ± *SEM*. Significance was assessed by two‐tailed Student's *t* test. *n* = 6, ***p < 0.001

To determine the potential role of ROCK1 in AD, firstly, we assessed ROCK1 activity by kinase assay in both frontal cortex and hippocampus of 8‐month‐old APP/PS1 mice and found that ROCK1 activity was markedly increased in AD mouse brain compared to age‐matched nontransgenic wild‐type (WT) control mice (Figure 1a), and secondly, the alterations successfully replicated in the brain of AD patients and suggested that the level of ROCK1 activity in AD brain was increased (Figure 1a). To explore the reason of ROCK1 activation, we found that in the brain of AD patients, ROCK1 mRNA was positively correlated with amyloid pathology‐associated CERAD scores (Figure S1), and the results obtained are consistent with previous observation (Henderson et al., 2016). In the brain of APP/PS1 mice, ROCK1 mRNA was also increased relative to WT mice (Figure S2A). Those results suggested that amyloid production may trigger ROCK1 transcription in AD. To reveal the mechanism of dysregulated ROCK1 transcription, we focused on ROCK1‐associated transcription factors. Previous studies demonstrated that transcription factors SP1 and SP6 were involved in ROCK1 gene transcription (Citron, Dennis, Zeitlin, & Echeverria, 2008; Yanuaryska et al., 2014; Zhang et al., 2016; Figure S2B). Therefore, we investigated the role of SP1 and SP6 in AD and their regulation of ROCK1 expression. With Western blot and immunostaining, both SP1 and SP6 were up‐regulated in the brain of APP/PS1 mice (Figure S2C,D). To explain the mechanism of SP1‐ and SP6‐mediated ROCK1 regulation in AD, we treated PC12 cell with Aβ40 and found that Aβ40 activated SP1 and SP6 expression as well as increased ROCK1 level (Figure S2E). Additionally, siRNA knockdown of either SP1 or SP6 could decrease ROCK1 expression (Figure S2F,G). Those results suggested that ROCK1 activation in AD was associated with amyloid pathology and dependent on up‐regulation of SP1 and SP6.

To further explore the association of ROCK1 and amyloid production, immunostaining analysis revealed that APP was specifically co‐localized with ROCK1 in the brain of APP/PS1 mice (Figure 1b). Next, to investigate whether ROCK1 interacts with APP in the context of AD, we found a marked increase in the interaction between ROCK1 and APP in cortical and hippocampal neurons of APP/PS1 mice compared to wild‐type (WT) mice via a novel application for PLA (Figure 1c). Then, we successfully validated our finding in postmortem brains from patients with AD via PLA (Figure 1d and Figure S3). Therefore, the results support that ROCK1 is activated and that its interaction with APP is up‐regulated in AD brain.

### 3.2 ROCK1 phosphorylates APP^695^ at Ser655

**Figure 2:**
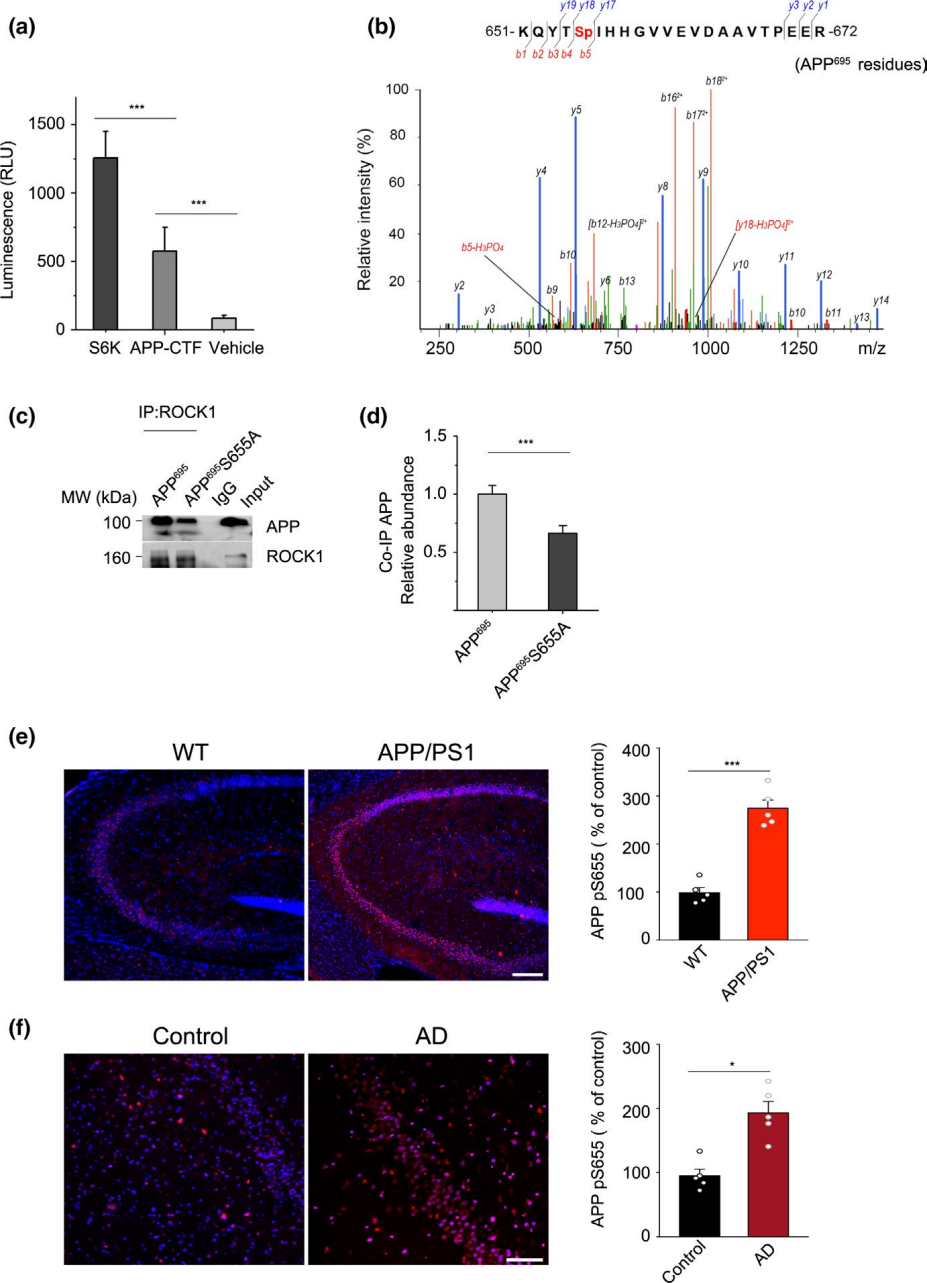
ROCK1 phosphorylates APP695 at Ser655. (a) ROCK1 activity assay when recombinant ROCK1 protein was incubated with S6K, APP‐CTF, and vehicle. One‐way ANOVA followed by Student–Newman–Keuls test, *F*_3,28_ = 6.28. (b) Representative S655 phospho‐peptide spectrum of APP^695^. APP‐CTF was incubated with recombinant ROCK1 and examined by LC‐MS/MS after 3 hr. (c) Co‐IP experiments in HEK 293T cells confirmed decreased interaction of ROCK1 with APP after S655A mutation in APP^695^ plasmid. (d) Quantification of interaction between ROCK1 and APP after APP695 and S655A plasmid transfection in HEK 293T cells. *t*_4_ = 6.67, two‐tailed Student's *t* test. (e) Immunofluorescence and quantification of phosphorylated APP at S655 (APP pS655) in the hippocampus of APP/PS1 and WT mice (*n* = 6, *t*_10_ = 8.72). Scale bar, 50 μm. (f) Immunofluorescence and quantification of phosphorylated APP at S655 (APP pS655) in the hippocampus of AD patients and normal control (*n* = 6, *t*_10_ = 5.8). Scale bar, 50 μm. Data were presented as Mean ± *SEM*. ***p < 0.001; *p < 0.05

To determine whether ROCK1 directly regulates APP through phosphorylation, recombinant active ROCK1 and APP C‐terminal fragment were analyzed in an in vitro kinase assay. As a positive control, we performed the kinase assay of ROCK1 with a known substrate, S6K. Notably, ROCK1 demonstrated kinase activity when incubated with APP‐CTF as the substrate (Figure 2a). To identify the phosphorylation sites on APP‐CTF by ROCK1, an in vitro reaction was performed using purified APP‐CTF and recombinant active ROCK1 protein and subsequently assessed by mass spectrometry. We identified a single phosphorylation site at Ser655 (S655) of APP^695^ (Figure 2b). To confirm the importance of this site for interaction, we repeated co‐IP with ROCK1 using mutant form APP^695^ S655A and found that co‐IP was decreased (Figure 2c,d). This result suggested that this mutation may disturb the interaction between ROCK1 and APP.

To reveal whether APP phosphorylation at the ROCK1‐targeted serine exists in AD brain, we developed a neo‐epitope‐specific antibody targeting APP S655 phosphorylation (Figure S4). Then, we identified that this new APP phosphorylation event occurs in the brains of AD mouse model and patients using the antibody. Notably, the level of APP S655 phosphorylation was increased in the brain of APP/PS1 mice and AD patients compared to control (Figure 2e,f). Hence, we identified that APP is a substrate of ROCK1 and APP phosphorylation at S655 was increased in AD.

### 3.3 ROCK1 promotes APP amyloidogenic processing

**Figure 3:**
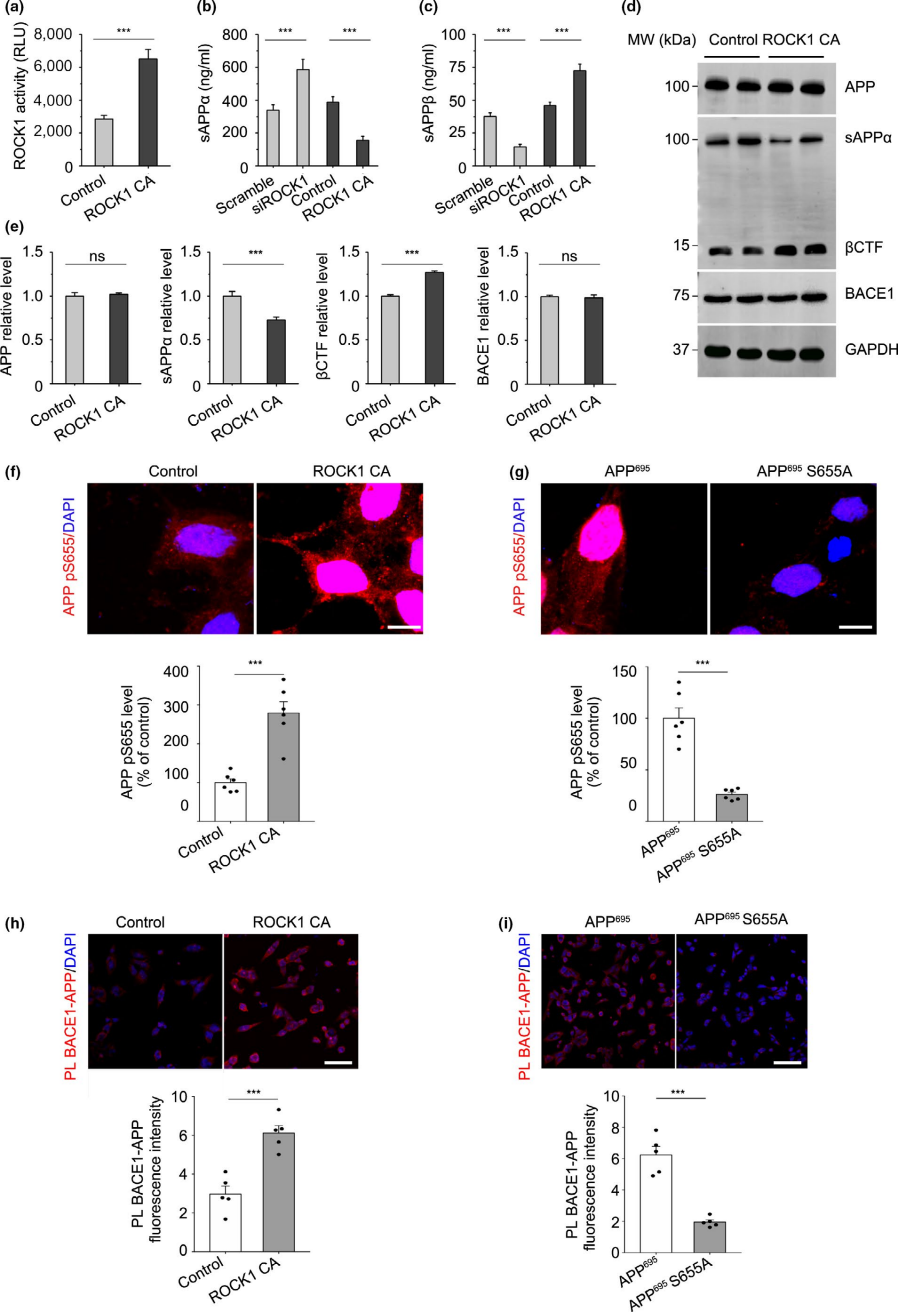
ROCK1 promotes APP amyloidogenic processing. (a) ROCK1 activity increased after ROCK1 CA plasmid transfection relative to control in HEK 293T APPsw cells by kinase assay (*t*_18_ = 4.48). (b) The levels of sAPPα analyzed by ELISA kit after 72 hr following ROCK1 CA plasmid and siROCK1 transfection in HEK 293T APPsw cells (*F*_3,30_ = 5.3). (c) The levels of sAPPβ were analyzed by ELISA after 72 hr following ROCK1 CA plasmid and siROCK1 transfection in HEK 293T APPsw cells (*F*_3,30_ = 7.7). (d) After ROCK1 CA plasmid transfection in HEK 293T APPsw cells, 72 hr later, cell lysates were prepared and APP (full‐length, APP‐FL), sAPPα, βCTF, and BACE1 were analyzed by Western blot. GAPDH was used as loading control. (e) Quantification of APP, sAPPα, βCTF, and BACE1 expression after ROCK1 CA plasmid transfection. Relative ratio to GAPDH was calculated by densitometry analysis. (f) Immunofluorescence and quantification of phosphorylated APP at S655 (APP pS655) after ROCK1 CA plasmid transfection relative to control in HEK 293T APPsw cells (*n* = 6, *t*_10_ = 5.88). Scale bar, 5 μm. (g) Immunofluorescence and quantification of phosphorylated APP at S655 (APP pS655) after APP695 and S655A plasmid transfection in HEK 293T cells (*n* = 6, *t*_10_ = 7.12). Scale bar, 5 μm. (h) Proximity ligation and quantification of PLA signal between BACE1 and APP after ROCK1 CA plasmid transfection relative to control in HEK 293T APPsw cells (*n* = 5, *t*_8_ = 7.62). Scale bar, 20 μm. (i) Proximity ligation and quantification of PLA signal between ROCK1 and APP after APP^695^ and S655A plasmid transfection in HEK 293T cells (*n* = 5, *t*_8_ = 6.11). Scale bar, 20 μm. Data were presented as Mean ± *SEM*. Two‐tailed unpaired Student's* t* test. ***p < 0.001

To investigate whether APP phosphorylation induced by ROCK1 affects α‐ and β‐secretase‐mediated cleavage pathway balance, a cell model over‐expressing ROCK1 (transfected with myc‐tagged ROCK1 plasmid, ROCK1 CA) was constructed in HEK293T cells with steadily expressed Swedish mutant APP^695^. Kinase activity assays confirmed that enhanced ROCK1 activity occurred in ROCK1 over‐expressing cells (Figure 3a). Activation of ROCK1 in these cells showed significant reduction of sAPPα production and an increase in sAPPβ levels (Figure 3b,c), while both full‐length APP and BACE1 levels were unchanged between two groups (Figure 3d,e). Meanwhile, inhibition of ROCK1 via siRNA increased sAPPα levels and decreased sAPPβ levels (Figure 3b,c). As above‐mentioned, the results suggest that ROCK1 activation promotes amyloidogenic processing of APP, and we proposed that phosphorylated APP induced by ROCK1 rather than nonphosphorylated one might be a better substrate for BACE1.

To validate this hypothesis, we investigated the effects of ROCK1‐induced APP phosphorylation on APP amyloidogenic processing in HEK293T APPsw cells. Firstly, we showed that activation of ROCK1 enhanced APP^695^ S655 phosphorylation while APP^695^ S655A mutant decreased APP^695^ S655 phosphorylation (Figure 3f,g). In addition, we found that ROCK1 activation promoted BACE1‐APP interaction and probably resulted in an accelerated catalytic reaction rate of APP processing by BACE1 via PLA (Figure 3h). Moreover, in cells transfected with APP^695^ WT or APP^695^ S655A, the phospho‐null mutant APP decreased BACE1‐APP interaction and APP processing significantly (Figure 3i). Accordingly, these studies demonstrated that APP phosphorylation by ROCK1 exacerbates amyloidogenic APP metabolism by promoting BACE1‐APP interaction and a higher catalytic reaction rate of BACE1‐mediated APP processing.

### 3.4 Blockade of APP S655 phosphorylation ameliorates amyloid pathology

**Figure 4:**
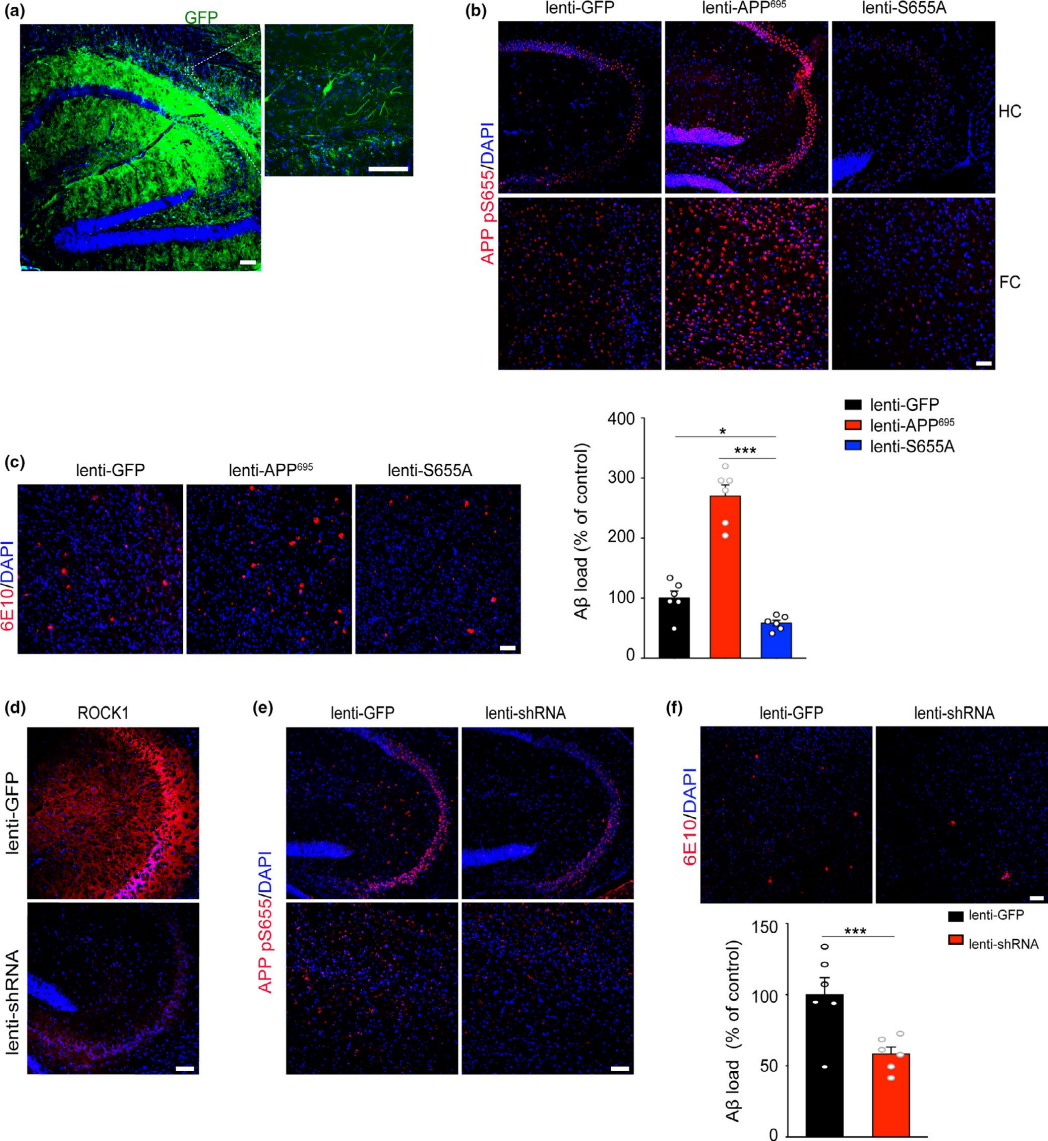
Blockade of APP S655 phosphorylation ameliorates amyloid pathology. (a) Microphotographs of APP/PS1 brain sections injected with virus and GFP were used to visualize viral diffusion. Scale bar, 50 μm. (b) Immunofluorescence of phosphorylated APP at S655 (APP pS655) after virus injection in APP/PS1 mice. Scale bar, 50 μm. (c) Immunofluorescence and quantification of Aβ plaque burden in the brains of APP/PS1 after virus injection (*F*_2,16_ = 8.12). Scale bar, 50 μm. (d) Immunofluorescence of ROCK1 in the brains of APP/PS1 mice after virus injection. Scale bar, 50 μm. (e) Immunofluorescence of phosphorylated APP at S655 (APP pS655) after virus injection in APP/PS1 mice. Scale bar, 50 μm. (f) Immunofluorescence and quantification of Aβ plaque burden in the brains of APP/PS1 after virus injection (*t*_10_ = 4.72). Scale bar, 50 μm. ***p < 0.001; *p < 0.05

To determine the in vivo role of APP phosphorylation in the AD model, we generated GFP‐tagged lentiviral vectors expressing APP^695^ WT (lenti‐APP^695^) and APP^695^ S655A (lenti‐S655A; Figure 4a). The viruses were stereotactically injected into the third ventricle of 5‐month‐old APP/PS1 mice. Control APP/PS1 littermates were injected with only GFP‐expressing lentivirus (lenti‐GFP). Two months after injection, these mice were evaluated by pathological examination. In immunohistochemical analysis with brain sections, APP^695^ WT over‐expressing APP/PS1 mice had increased APP phosphorylation at S655 compared to control‐vector‐injected mouse brain. S655A mutant presented a lower level of APP S655 phosphorylation than lenti‐APP^695^ WT mice (Figure 4b). Immunofluorescence staining with 6E10 showed Aβ plaque deposits were increased in APP^695^ WT over‐expressing mice compared to the control group. However, the amyloid pathology was significantly alleviated in the brain of S655A‐expressing mice (Figure 4c). Collectively, these results support that a nonphosphorylated mutant APP S655A attenuates amyloid pathology in APP/PS1 mice.

Further, to ascertain whether ROCK1 knockdown could attenuate APP S655 phosphorylation and amyloid pathology in the AD mouse model, we injected lentiviral‐expressing ROCK1 shRNA vectors (lenti‐shRNA) into the third ventricle of 5‐month‐old APP/PS1 mice. Two months after intracerebral injection, decreased expression level of ROCK1 was confirmed in the brain compared with scramble shRNA‐injected control group (Figure 4d). Down‐regulation of ROCK1 reduced APP phosphorylation at S655 and the density of amyloid plaques in the brain (Figure 4e,f). These findings suggest that shRNA knockdown of ROCK1 could decrease APP S655 phosphorylation and attenuate amyloid pathology.

### 3.5 ROCK1 inhibition improves cognitive behaviors in APP/PS1 mice

**Figure 5:**
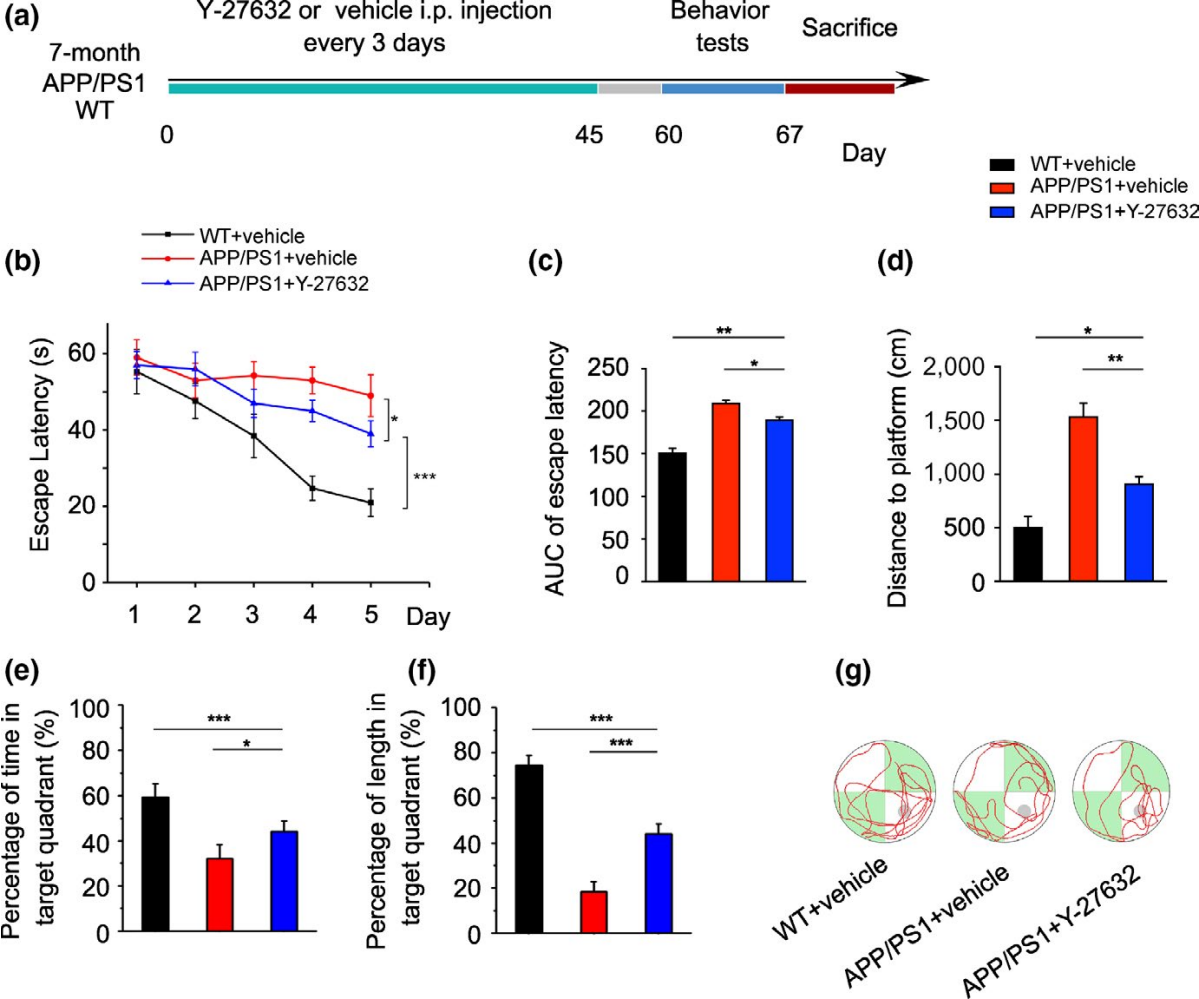
ROCK1 inhibition improves cognitive behaviors in APP/PS1 mice. (a) The graph shows the experimental scheme. Y‐27632 or vehicle was injected intraperitoneally in APP/PS1 and WT littermates every 3 days for 45 days. After 60 days, cognitive behaviors were examined. (b) Escape latency to find the hidden platform in MWM test during the 5‐day training and learning regimen. Two‐way ANOVA followed by Student–Newman–Keuls post hoc analysis (group effect: *F*_2,75_ = 59.60; time effect: *F*_4,75_ = 41.18; group × time effect: *F*_8,75_ = 9.17, *p* < 0.001). (c) Area under curve (AUC) of escape latency in the training trials (two‐way ANOVA followed by Student–Newman–Keuls post hoc analysis, *F*_2,8_ = 54.95). (d) Swimming distance to find the platform in on Day 5 is shown (one‐way ANOVA followed by Student–Newman–Keuls post hoc analysis *F*_2,15_ = 12.85). (e) The percentage of time in the target quadrant in the probe trials is given (*F*_2,34_ = 3.55). (f) The percentage of swimming distance occurring within the target quadrant is calculated for the probe trials (*F*_2,34_ = 4.83). (g) Representative swim paths of individual mice with indicated genotypes. The gray circle indicates the hidden platform. Data are presented as mean ± *SEM*. *n* = 5–7 per group. ***p < 0.001; **p < 0.01; *p < 0.05

To further explore the therapeutic role of ROCK1 inhibition, we adopted a ROCK1 inhibitor Y‐27632 for treatment of APP/PS1 mice and WT control. Because APP/PS1 mice showed a rapid formation of amyloid plaques from 6 to 8 months (Yan et al., 2009), Y‐27632 treatment was delivered at 7 months for 45 days. Injection with saline only was performed on control (Figure 5a). The Morris water maze (MWM) test was conducted 2 weeks after the end of treatment. There was no difference on swimming speed in each group (Figure S5A). APP/PS1 mice showed a longer latent period, shorter time, and path length in the target quadrant compared to WT mice (Figure 5b–g and Figure S5B–D). Furthermore, overall improved spatial learning and memory was detected after Y‐27632 treatment in APP/PS1 mice. Y‐27632‐treated AD mice showed a short latent period, a clear preference for the target quadrant, and more successful trials compared to the saline‐injected controls. Moreover, there was a significant effect of group on proximity with Y‐27632‐treated APP/PS1 mice relative to the WT group, suggesting a partial rescue of ROCK1 inhibition for APP/PS1 mice (Figure 5b–g). These data suggest that a beneficial effect of Y‐27632 occurs to ameliorate learning and memory deficits in APP/PS1 mice.

### 3.6 ROCK1 inhibition decreases APP amyloidogenic metabolism in vivo

**Figure 6:**
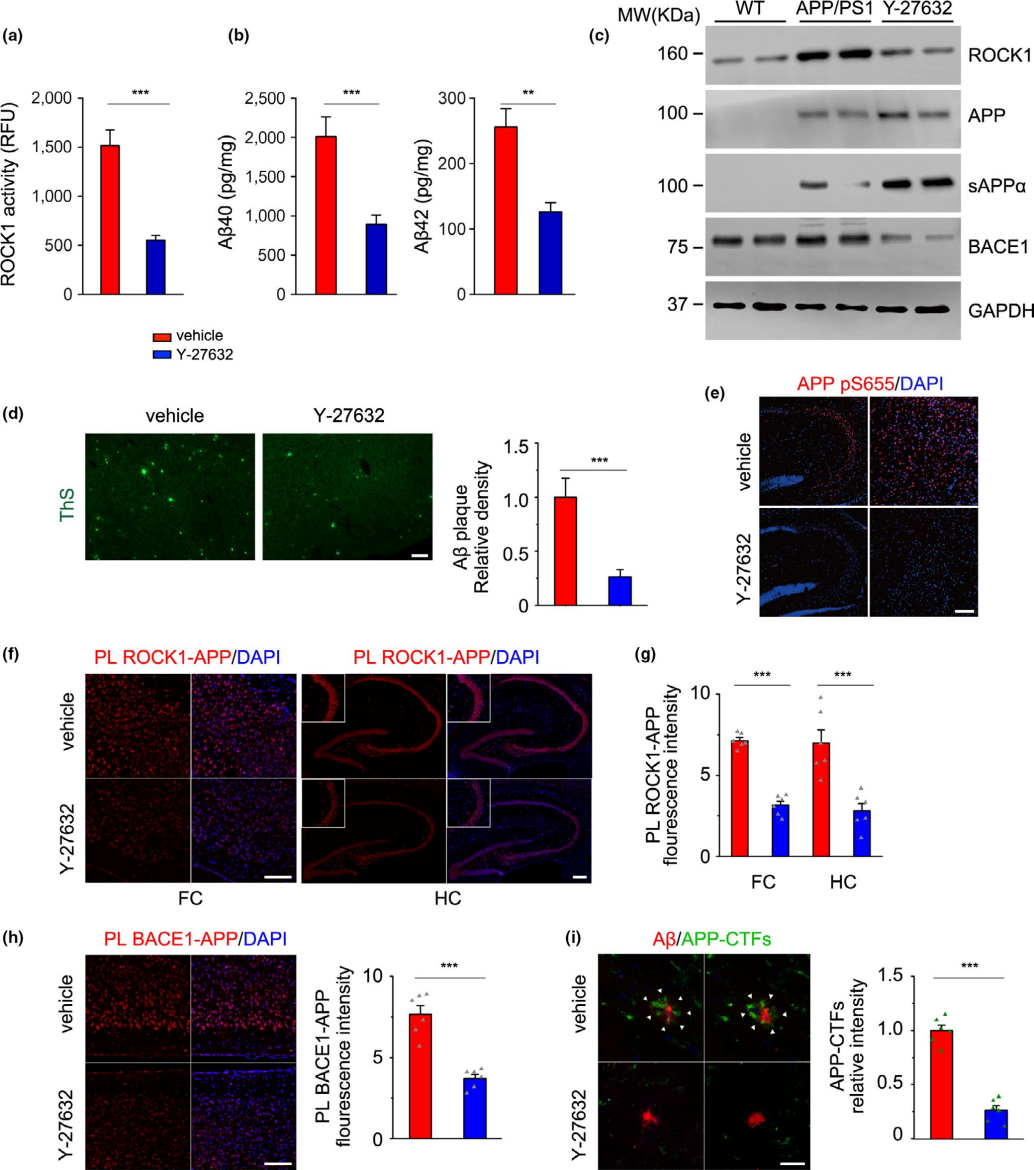
ROCK1 inhibition decreases APP amyloidogenic metabolism and amyloid pathology in vivo*.* Mouse brain homogenates were prepared from 8‐month‐old APP/PS1 and WT mice after Y‐27632 treatment. (a) ROCK1 activity was decreased in the brain of APP/PS1 mice after Y‐27632 treatment as measured by kinase assay (*t*_8_ = 5.87). (b) Aβ40 and Aβ42 levels (*t*_8_ = 4.71, *t*_8_ = 6.13) in mouse brain homogenates were analyzed by ELISA. (c) ROCK1, APP, sAPPα, and BACE1 in mouse brain homogenates were analyzed by Western blot. GAPDH was used as internal control to normalize the calculated abundance of the targeted proteins. (d) Immunofluorescence and quantitative analysis of Aβ plaque burden in the brains of APP/PS1 mice after Y‐27632 treatment (*t*_10_ = 5.16). Scale bar, 50 μm. (e) Immunofluorescence of phosphorylated APP at S655 (APP pS655) after Y‐27632 treatment. Scale bar, 50 μm. (f) Proximity ligation between ROCK1 and APP in the frontal cortex (FC) and hippocampus (HC) of APP/PS1 mice after Y‐27632 treatment. Scale bar, 50 μm. (g) Quantitative analysis of PL signals between ROCK1 and APP in the FC (*t*_10_ = 12.79) and HC (*t*_10_ = 6.75) of APP/PS1 mice after Y‐27632 treatment. (h) Proximity ligation between BACE1 and APP in the cortex of APP/PS1 mice after Y‐27632 treatment and quantitative analysis of the PL signals (*t*_10_ = 6.75). Scale bar, 50 μm. (i) Immunofluorescence of Aβ plaque (red) and APP‐CTFs (green) and quantitative analysis of APP‐CTFs in the diffuse part of amyloid plaques (*t*_10_ = 11.06). Scale bar, 20 μm. Data are presented as mean ± *SEM*. *n* = 5–7 per group; Two‐tailed unpaired Student's *t* test and one‐way ANOVA followed by Student–Newman–Keuls post hoc analysis. ***p < 0.001; **p < 0.01; *p < 0.05

Amyloid precursor protein fragments derived from β‐secretase‐mediated APP processing and amyloid plaque formation are driving forces inducing neurodegeneration and memory impairment (Lauritzen et al., 2016; Turner, O'Connor, Tate, & Abraham, 2003). Next, we investigated whether ROCK1 inhibition‐induced memory restoration reflected biochemical and neuropathological amelioration. A kinase assay confirmed that ROCK1 activity in the brains of APP/PS1 mice was down‐regulated following Y‐27632 treatment (Figure 6a). To investigate the effects of ROCK1 inhibition in APP processing, we detected APP cleavage fragments in the brain with Western blot. Compared to saline‐treated APP/PS1 mice, sAPPα production was increased and βCTF was decreased significantly in Y‐27632‐treated APP/PS1 mice, with ROCK1 levels down‐regulated (Figure 6b,c and Figure S6). Moreover, it is notable that BACE1 levels were also reduced after 45‐day treatment, possibly resulting from its substrate down‐regulation, that is, ROCK1‐phosphorylated APP at Ser655. Enzyme‐linked immunosorbent assay results indicated that Aβ40 and Aβ42 levels were decreased in brains of Y‐27632‐treated mice (Figure 6b). Thus, we have demonstrated that ROCK1 inhibition represses APP amyloidogenic processing in APP/PS1 mice.

### 3.7 ROCK1 inhibition ameliorates amyloid pathology in APP/PS1 mice

To further confirm ROCK1‐inhibition‐induced decreases β‐processing of APP, immunofluorescence analysis of brain sections was examined and exhibited an overall reduction of amyloid plaque formation and APP S655 phosphorylation in both the hippocampus and cortex of ROCK1‐inhibited mice (Figure 6d,e). Moreover, PLA of ROCK1‐APP in brain of Y‐27632‐treated mice showed a marked decrease in interaction of ROCK1 with APP resulting from inhibition of ROCK1 activity (Figure 6f,g). Another PLA for BACE1‐APP interaction suggested that the significant reduction of reaction rate for BACE1‐mediated APP processing, inducing β‐CTF decrease and an increase in sAPPα following ROCK1 inhibition, stems from a loss in interaction (Figure 6h). To further assess the structure and composition of senile plaques, we used immunofluorescence analysis. Results revealed co‐labeling of Aβ and APP‐CTFs in amyloid plaques (Figure 6i). Aβ aggregated in the cores of plaques, and APP‐CTFs were found in the surrounding halo and thus have a potential role in Aβ accumulation and plaque growth (Willem et al., 2015). A reduced signal for APP‐CTF peptides was obtained in the diffuse part of amyloid plaques from inhibitor‐treated mouse brain sections. Since ROCK1 inhibition reduced BACE1‐derived β‐CTF, it is concluded that β‐CTF decrease is the key contributor preventing amyloid formation and involved in ameliorating cognitive deficits in Y‐27632‐treated APP/PS1 mice.

## 4 DISCUSSION

In the present study, we firstly have identified ROCK1 as a novel kinase phosphorylating the APP ectodomain at Ser655 and ROCK1 was activated in AD brain. We further demonstrated that ROCK1‐mediated APP phosphorylation at S655 promotes amyloidogenic processing of APP by increasing interaction of BACE1 with APP. Blockade of ROCK1 activity was protected against both APP‐CTF and Aβ accumulation and improved learning and memory in APP/PS1 mice. Hence, ROCK1‐induced APP S655 phosphorylation in AD significantly promotes amyloidogenic APP processing and subsequently leads to cognition deficits during the progression of AD.

Rho‐associated coiled‐coil kinase 1 acts as a downstream effector in the intracellular signaling of several G protein‐coupled receptors, and ROCK1 kinase is involved in a wide range of pathological conditions (Homan & Tesmer, 2015; Loirand, 2015). Our findings reveal that ROCK1 activity is increased in brain tissues from both AD patients and in an AD mouse model, and the results are consistent with previous observations that up‐regulation of ROCK1 mRNA and protein is associated with neurodegenerative diseases such as HD and Parkinson's disease (PD; Narayanan, Chopra, Rosas, Malarick, & Hersch, 2016; Villar‐Cheda et al., 2012). To explore the mechanism of ROCK1 activation, we found that ROCK1 mRNA in the AD brain was positively correlated with amyloid pathology‐associated CERAD scores, and proposed that dysregulated transcription of ROCK1 may be associated with its activation. We confirmed that ROCK1‐associated transcription factors, SP1 and SP6, were associated with amyloid production and up‐regulated in AD mouse model and those results were consistent with previous reports (Citron et al., 2008).

Given the findings of relations of ROCK1 with APP and Aβ, we investigate the possible link between ROCK1 activation and APP post‐translational modification (PTM). Post‐translational modification of APP has been a focus of research in the field of AD, including examinations of APP glycosylation, ubiquitination, and phosphorylation (Marcelli et al., 2017). These modifications have been proposed as regulatory mechanisms to affect APP trafficking and cleavage, signal transduction, and axonal outgrowth (Ahmed, Zahid, Mahboob, & Farhat, 2017; Ando, Iijima, Elliott, Kirino, & Suzuki, 2001; Schedin‐Weiss, Winblad, & Tjernberg, 2014). Since APP processing and Aβ production involve intracellular fragment transport and vesicle trafficking, APP processing can be regulated via modulating its phosphorylation. For example, phosphorylation of APP at Thr668 in the motif ^667^VTPEER^672^ has been reported to regulate cell cycle progression, axonal outgrowth, and translocation of AICD to the nucleus (Müller, Meyer, Egensperger, & Marcus, 2008). But in the most highly conserved ^654^YTSI^656^ motif of the APP ectodomain, S655 only was reported to be phosphorylated by PKC and the effect on function remained unknown (Lane, Gatson, Small, Ehrlich, & Gandy, 2010). However, except for the phosphorylation of PKC, there may be other specific kinases to involve in the signal transductions, especially for AD pathology. Here, we found it and confirm that ROCK1 activation promotes the interaction of ROCK1 with APP and APP phosphorylation in AD. Rho‐associated coiled‐coil kinase 1 phosphorylated APP at Ser655, regulating its amyloidogenic processing. Meanwhile, we found that decreased APP phosphorylation at S655 via expression of nonphosphorylated mutant APP S655A and interaction of ROCK1 with APP results in decreased production of BACE1‐dependent APP proteolytic products and a reduction in amyloid burden. These results imply that ROCK1 activation has critical roles in facilitating the formation of well‐characterized AD senile plaque pathology.

Next, we need to answer whether the ROCK1 signal transduction cascade could play a role in treatment of the AD. Previous studies have shown that selective ROCK1 inhibitors can improve arterial relaxation and vascular remodeling (Boland et al., 2015; Green et al., 2015). Fasudil is the first approved ROCK inhibitor for clinical use in the treatment of cerebral vasospasm, ischemia‐induced brain damage, and pulmonary hypertension (Gupta et al., 2013; Olson, 2008). In the nervous system, ROCK1 pharmacological inhibition resulted in prevention of fostered axonal regeneration, beneficially regulated microglial dysfunction, and ameliorated neurodegeneration in models of PD and other neurodegenerative disorders (Borrajo, Rodriguez‐Perez, Villar‐Cheda, Guerra, & Labandeira‐Garcia, 2014). Additionally, several studies demonstrated that ROCK1 inhibition attenuated tau pathology, α‐synuclein, or HTT aggregation (Tatenhorst et al., 2016). In this study, we observed that ROCK1 inhibition led to partial rescue of spatial learning and memory impairments in AD mouse. This finding correlated with partial rescue of amyloid pathology. Furthermore, we confirmed the molecular mechanisms for it and ROCK1 inhibition decreased amyloid plaque formation in the brain of APP/PS1 mice and prevented APP‐CTF aggregation in the surrounding halo of amyloid plaques. Thus, series of evidences support that ROCK1 inhibition could be a promising target in developing a therapeutic strategy for these neurodegenerative disorders.

Based on these findings, we proposed that activated ROCK1 targets APP Ser655 phosphorylation, which then promotes amyloid processing and pathology. Inhibition of ROCK1 could be a potential therapeutic approach for AD (Figure S7). Recently, a series of previous clinical trials focused on Aβ production and clearance have failed (Rafii & Aisen, 2015). Our findings suggest that upstream intervention, for example, reduction of phosphorylated APP, rather than downstream intervention of this post‐translational modification, for example, regulating APP cleaving enzyme functions, could reduce amyloid deposition and aggregation. Hence, inhibition of ROCK1 kinase activity may be a novel therapeutic strategy targeting upstream APP processing for the treatment of AD.

## CONFLICT OF INTEREST

None declared.

## AUTHOR CONTRIBUTIONS

YB.H, RJ.R, and YF.Z performed the experiments, analyzed the data, and wrote the manuscript. Y.H analyzed the data and wrote the manuscript. HL.C, H.W, and PJ.C assisted in the mouse experiments. WY.Q and C.M provided critical materials. G.W and HZ.C designed all of the experiments, supervised the project, and edited the manuscript.

## Data Availability

Authors declare that the author provides the data to requester when there is a reasonable request for all data supporting the findings.
