# Supplementary material for: Rho‐associated coiled‐coil kinase 1 activation mediates amyloid precursor protein site‐specific Ser655 phosphorylation and triggers amyloid pathology
Source: Aging Cell. 2019 Jul 9;18(5):e13001. doi: 10.1111/acel.13001 (PMC6718535; doi:10.1111/acel.13001)
Supplement: Supplementary file 1 [file ACEL-18-e13001-s001.docx]

**ROCK1 activation mediates APP site-specific Ser655 phosphorylation and triggers amyloid pathology**

Authors: Yong-Bo Hu^1,2#^, Ru-Jing Ren^1#^, Yong-Fang Zhang^2#^, Yue Huang^3,4^, Hai-Lun Cui^1^, Chao Ma^5^, Wen-Ying Qiu^5^, Hao Wang^2^, Pei-Jing Cui^6^, Hong-Zhuan Chen^2,7*^, Gang Wang^1*^

**Supplementary Figures**

**
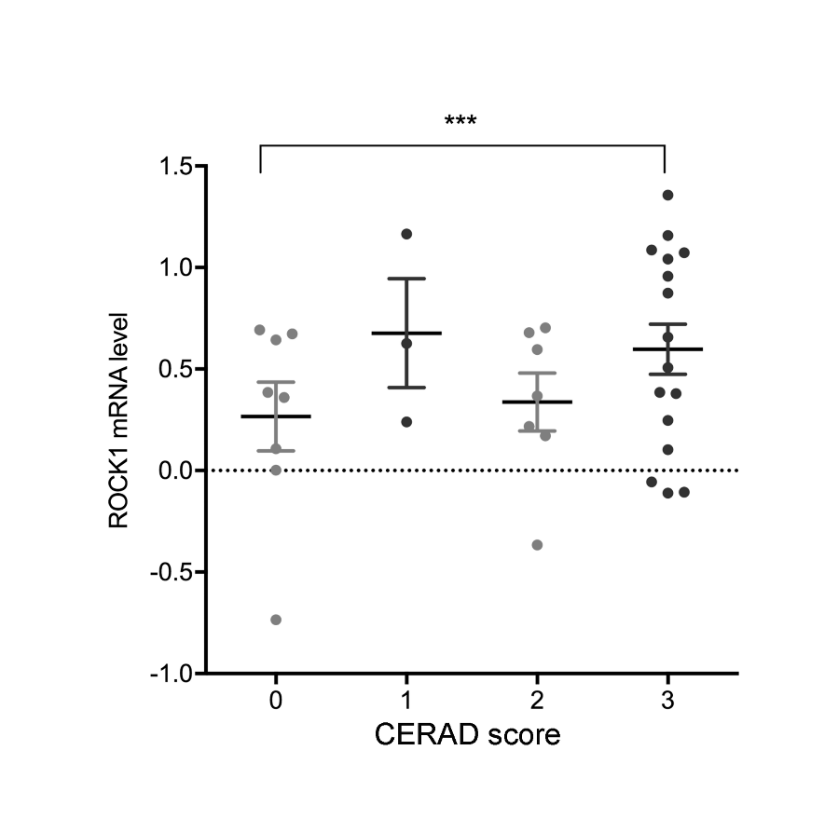
**

**Figure S1 ROCK1 mRNA was positively correlated with amyloid pathology-associated CERAD scores**

Data were presented as Mean ± SEM (n=34, *F_3,32_*=7.78). one-way ANOVA followed by Newman-Keuls post hoc analysis . ***p<0.001.

**
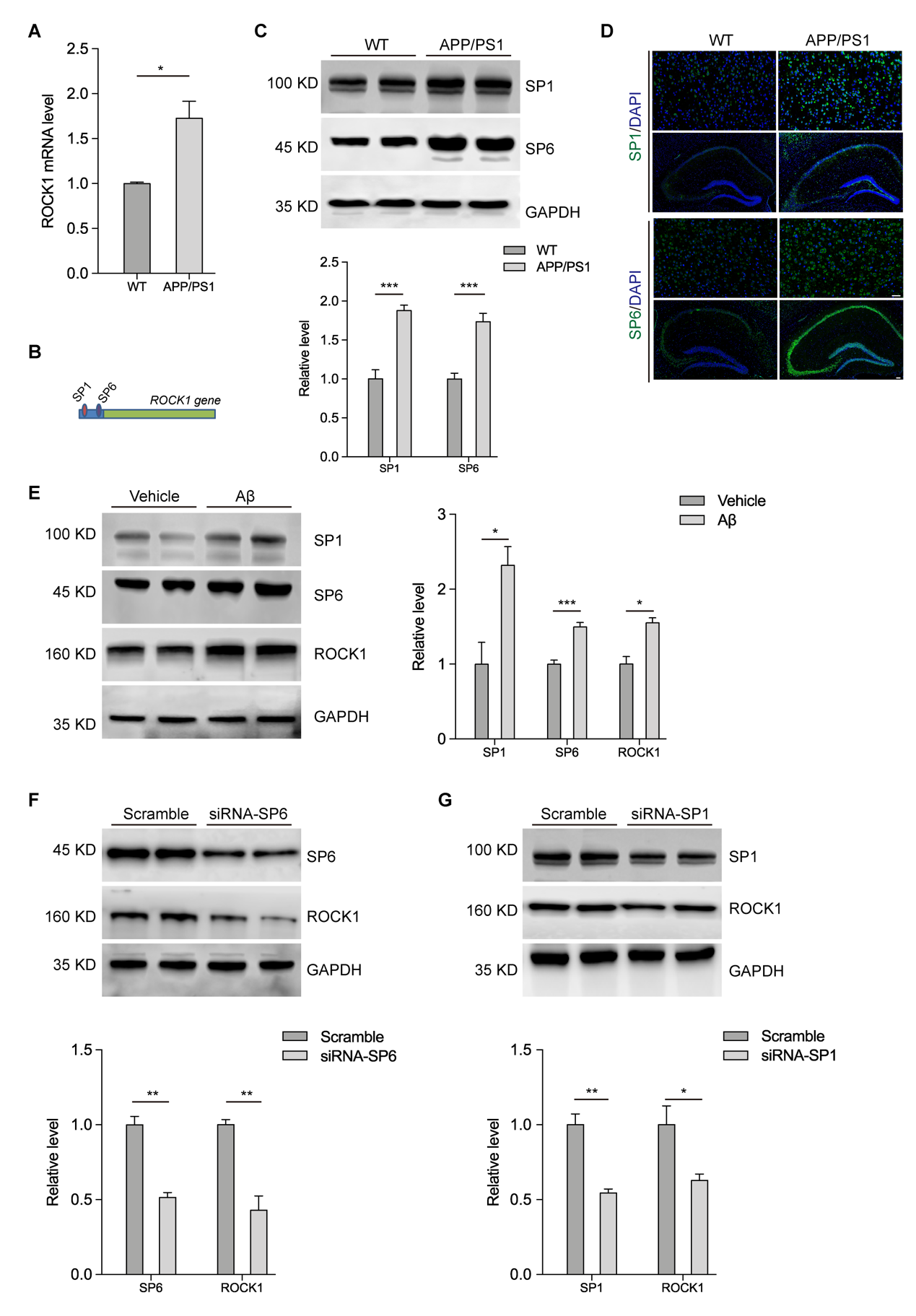
**

**Figure S2. Transcription factor SP1 and SP6 promoted activation of ROCK1 expression**

A. ROCK1 mRNA increased in the hippocampus of APP/PS1 mice by qPCR (*t_10_*=2.38).

B. Transcription factor SP1 and SP6 regulated ROCK1 transcription.

C. Up-regulation of SP1 (*t_4_*=6.49)and SP6 (*t_4_*=5.65) in the brain of APP/PS1 mice by Western blot.

D. SP1 and SP6 expression in the FC and HC of APP/PS1 and WT mice by  immunofluorescence. Scale bar, 50 μm.

E. Aβ increased SP1(*t_4_*=3.44), SP6(t_4_=6.19) and ROCK1 (*t_4_*=4.55) expression.

F. SP6 knockdown (*t_4_*=7.53) decreased ROCK1 expression (*t_4_*=5.68).

G.SP1 knockdown (*t_4_*=5.98) decreased ROCK1 expression (*t_4_*=2.81).

Data were presented as Mean ± SEM. Significance was assessed by two-tailed student’s *t* test. n=6, **p*<0.05, ***p*<0.01, ****p*<0.001.


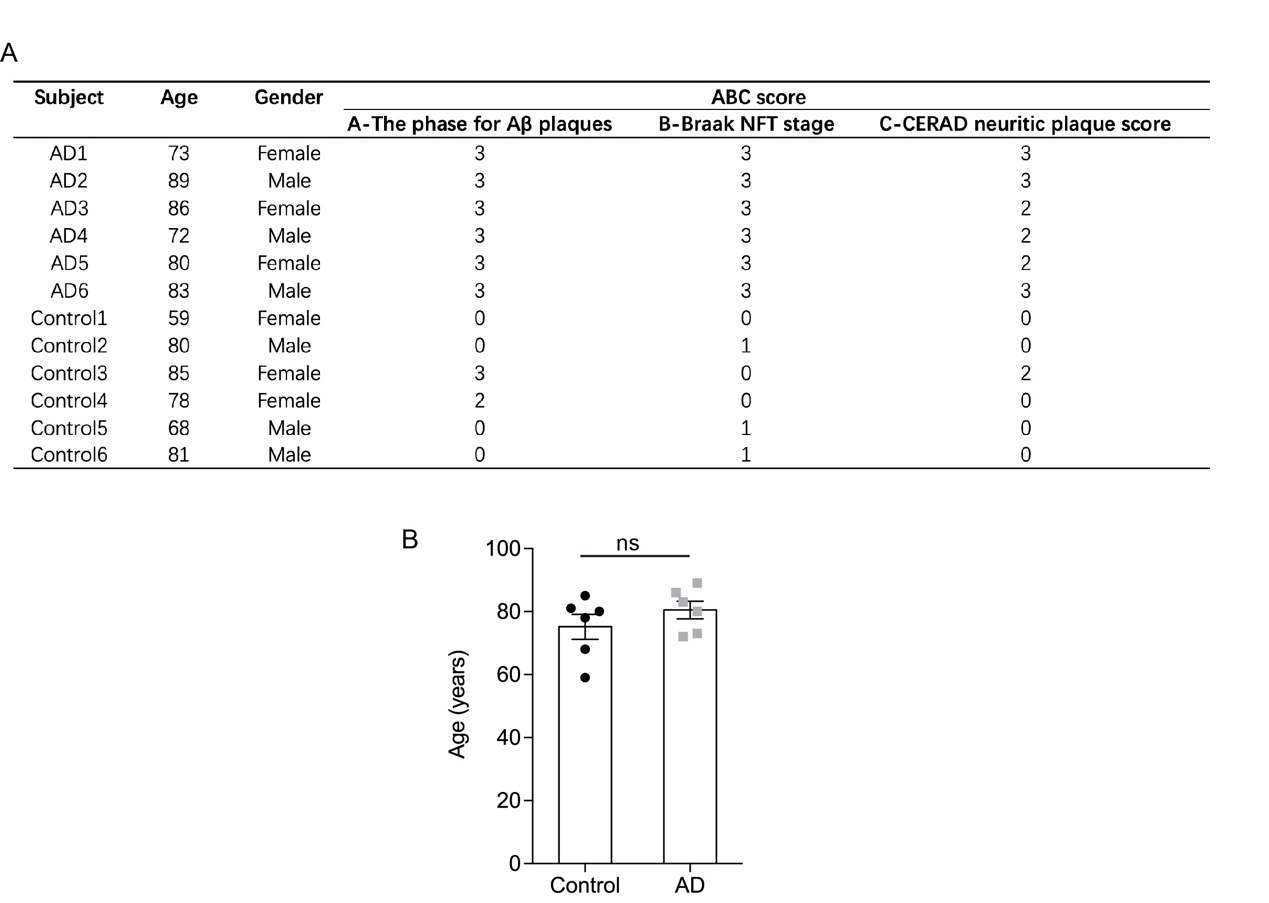


**Figure S3 Information of AD patients and control included in this study**

(A) Epidemiological aspects of AD patients and control.

(B) Age distribution in the groups of subjects . Data were presented as Mean ± SEM (n= 6 per group, two-tailed student’s *t* test, *t*_10_=1.1, ns, not significant.).


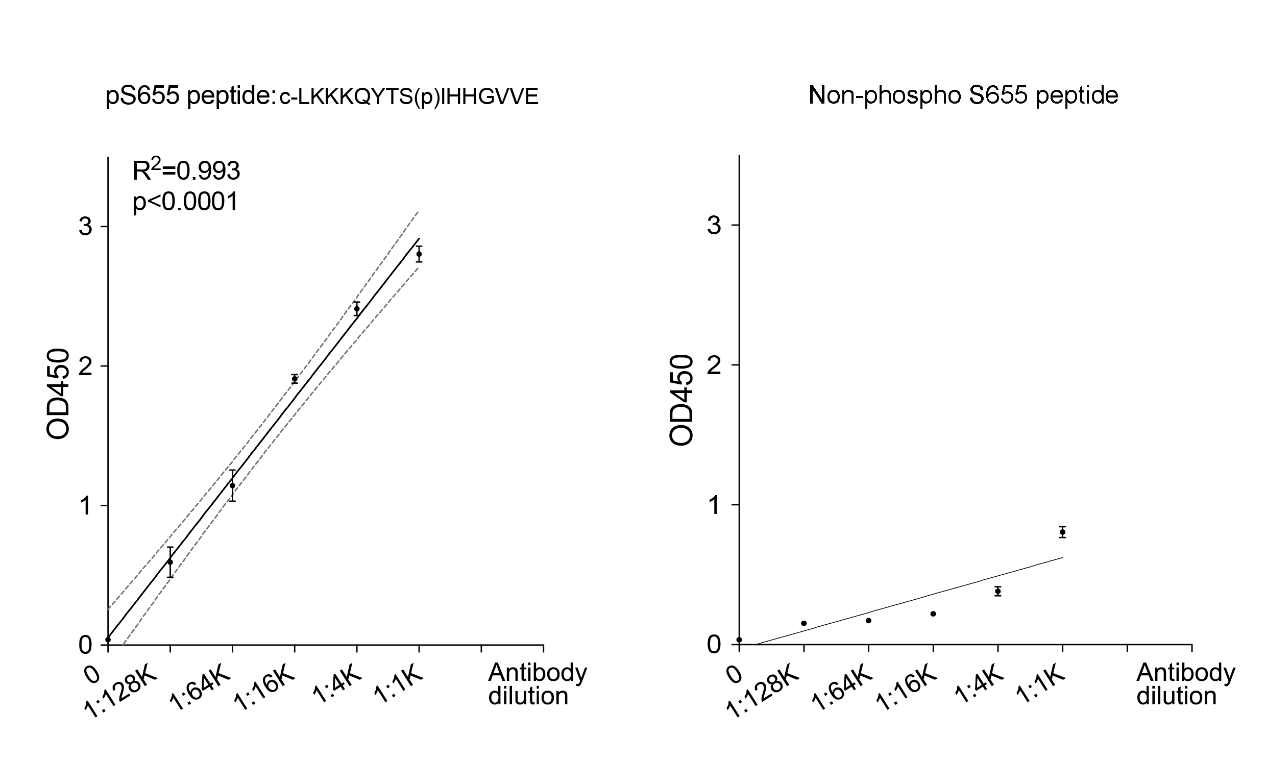


**Figure S4 Confirmatory data of APP pS655 antibody.**

ELISA assay of confirmatory data with phosphorylated APP S655 peptide and non-phosphorylated peptide.


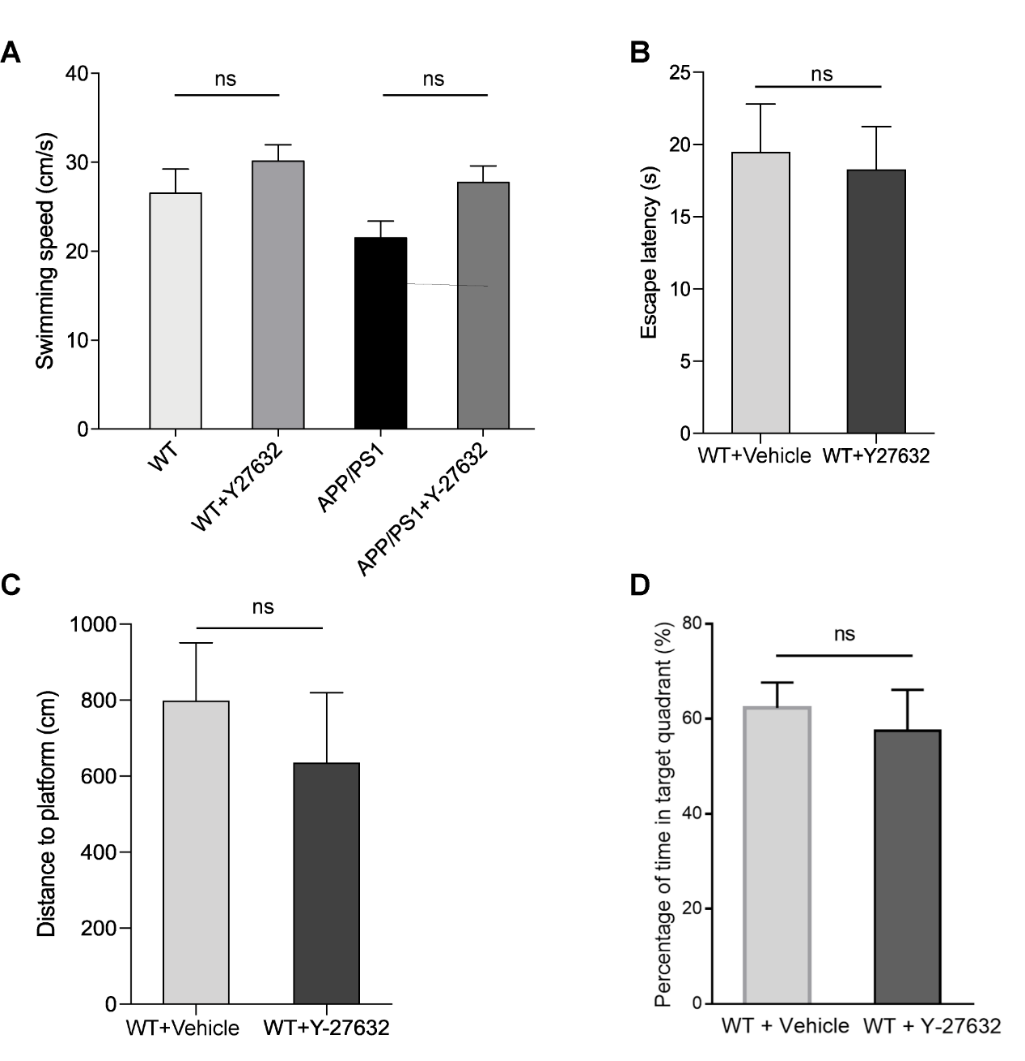


**Figure S5 WT mice were treated with Y-27632 every 3 days for 45 days**

1. Swimming speed of mice in different groups (*F_3,20_*=3).

(B) The escape latency to find the platform in Day5 of the training trails (*t_10_*=0.27).

(C) The swimming distance to find the platform on Day5 of the training trails (*t_10_*=0.68).

(D) The percentage of time in target quadrant in the probe trails (*t_18_*=1.64).

Data were presented as Mean ± SEM (n= 5-7 per group); Significance was assessed by two-tailed student’s *t* test. ns, not significant .


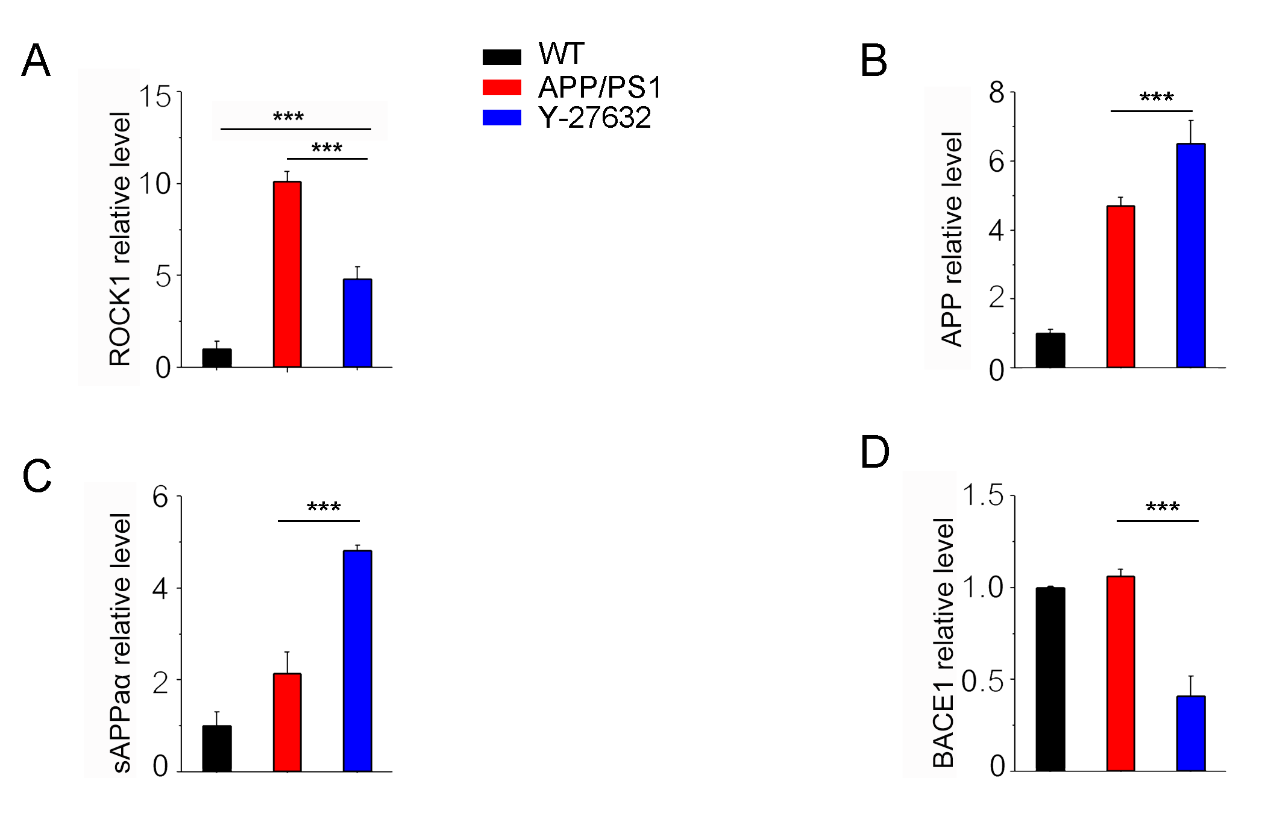


**Figure S6 Quantification of protein with western blot in mice after Y-27632 treatment**

A) Quantification of ROCK1 expression. Relative ratio (RR) to GAPDH was calculated by densitometry analysis (*F_2,12_*=90.58).

(B) Quantification of APP protein levels RR to GAPDH was calculated by densitometry analysis (*F_2,12_*=67.87).

(C) Quantification of sAPPα expression. RR to GAPDH was calculated by densitometry analysis (*F_2,12_*=46.01).

(D) Quantification of BACE1 expression. RR to GAPDH was calculated by densitometry analysis (*F_2,12_*=29.43).

Data were presented as Mean ± SEM (n= 5-7 per group); one-way ANOVA followed by Student-Newman-Keuls post hoc analysis. ****p*<0.001.


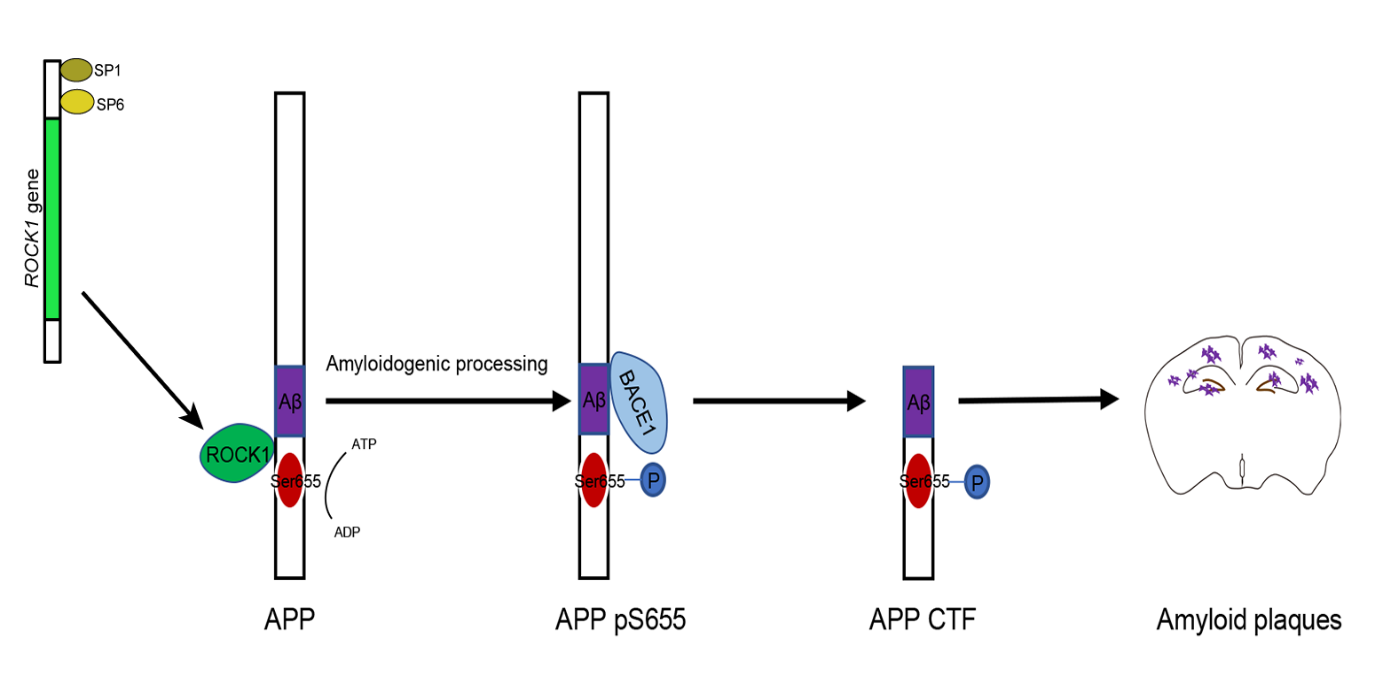


Figure S7 **Illustration of ROCK1 activation mediating APP site-specific Ser655 phosphorylation and triggering amyloid pathology**
